# Complete Response of Liver Metastasis of Gastric Cancer Treated by S-1 Chemoradiotherapy: A Case Report

**DOI:** 10.1155/2012/368428

**Published:** 2012-08-13

**Authors:** Tomonori Miyazawa, Kazuyu Ebe, Norihiko Koide, Nobuhiro Fujita

**Affiliations:** ^1^Department of Surgery, Joetsu General Hospital, Joetsu 943-8507, Japan; ^2^Department of Radiation Therapy, Joetsu General Hospital, Joetsu 943-8507, Japan

## Abstract

This paper presents a case of suspected liver metastasis of gastric cancer and a virtual complete response to S-1 chemoradiotherapy. A 69-year-old man underwent distal gastrectomy for gastric cancer in 2008. Multiple liver metastases occurred in 2009. He underwent 15 courses of S-1 therapy and radiation therapy (37.5 Gy). Abdominal computed tomography showed virtual complete disappearance of liver metastasis after chemoradiotherapy. Hence, this case was interpreted as a complete response. No sign of recurrence was noted 18 months after complete response was confirmed. S-1 chemoradiotherapy is likely to be effective in treating patients with liver metastases of gastric cancer.

## 1. Introduction

S-1 is an oral prodrug of fluorouracil (5-FU) with 2 biochemical modulators (gimeracil = 5-chloro-2, 4-dihydroxypyridine inhibiting 5-FU degeneration by dihydroxypyridine dehydrogenase, and oteracil = potassium oxonate which reduces the incidence of gastrointestinal toxicity by suppressing the activation of 5-FU in the gastrointestinal tract) [1]. The SPIRITS trial showed that in metastatic gastric cancer S-1 plus cisplatin is superior to S-1 alone and, therefore, is considered as a standard treatment for advanced gastric cancer [2].

However, the use of S-1 plus cisplatin should be carefully decided in elderly patients, and if deemed inappropriate, S-1 should be administered as a single agent [3]. The liver is a common site of metastasis of gastric cancer; however, the treatment for liver metastasis has not been yet established. Here, we report a case of liver metastases of gastric cancer that showed complete response (CR) to S-1 chemoradiotherapy.

## 2. Case Presentation

**Figure 1:**
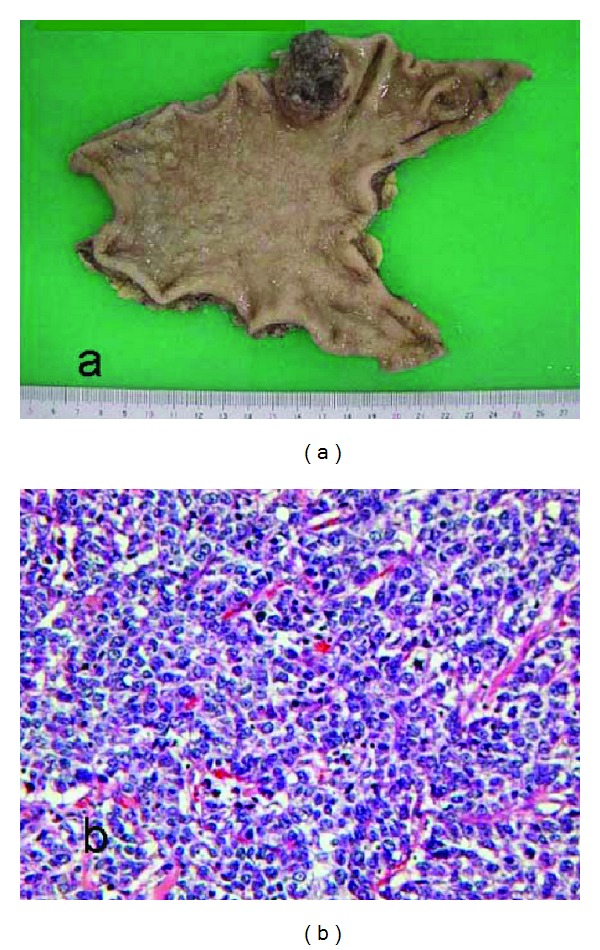
Resected specimen and pathological findings. (a) Macroscopic appearance of surgically resected specimen showing type 2 advanced gastric cancer in the corpus of the stomach. (b) Pathological examination showing a poorly differentiated adenocarcinoma (H&E stain).

**Figure 2:**
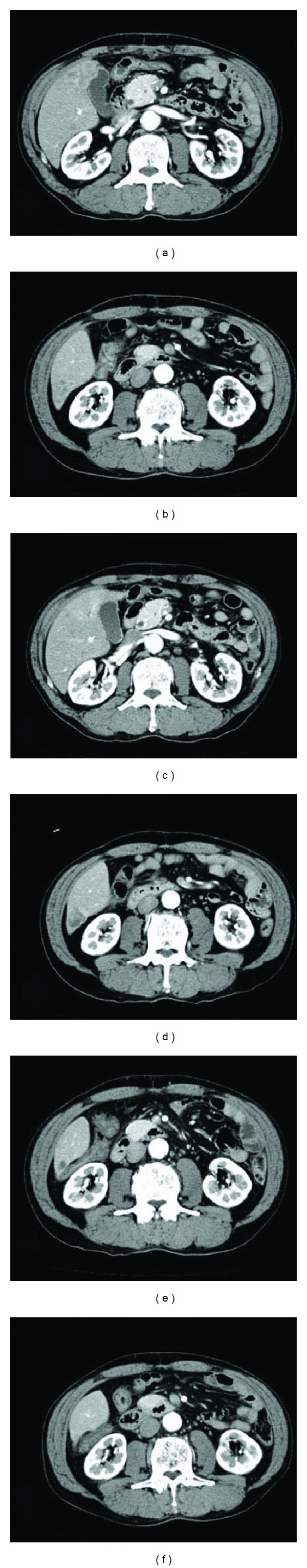
Abdominal computed tomography (CT) findings. (a)-(b) CT scan before chemoradiotherapy showing multiple liver metastases in S5 and S6. (c)-(d) CT scan after 5 cycles of S-1 administration showing disappearance of the tumor in S5, while tumor was still visible in S6. (e) CT scan after radiation and 10 cycles of S-1 administration showing reduction of tumor in S6. (f) CT scan after radiation and 15 cycles of S-1 administration showing disappearance of liver metastasis; hence, this case was interpreted as a complete response.

A 69-year-old man underwent distal gastrectomy for gastric cancer in 2008. Pathological examination showed a poorly differentiated adenocarcinoma invading the muscularis propria, without lymph node metastasis (T2a N0 M0/Stage 1B, Figures 1(a) and 1(b)). In 2009, an abdominal computed tomography (CT) scan showed multiple heterogeneous low-density masses in S5 and S6 of the liver (Figures 2(a)-2(b)). We diagnosed this as multiple liver metastases. The standard chemotherapy regimen for metastatic gastric cancer in Japan is S-1 plus cisplatin; however, in this case, a combination was considered inappropriate because the patient had mild renal dysfunction (creatinine clearance, 50 mL/min). We started S-1 administration (100 mg/twice daily on days 1–14, every 3 weeks) in July 2009. Abdominal CT after 5 cycles of S-1 revealed a virtual complete disappearance of the tumors in S5 but not of those in S6 (Figure 2(c)-2(d)). Hepatic radiation (37.5 Gy in 15 fractions) for the tumor in S6 was performed in May 2010. Abdominal CT after radiation and 10 cycles of S-1 showed reduction in the tumor masses in S6 (Figure 2(e)).

Another series of abdominal CT performed after radiation and 15 cycles of S-1 showed complete disappearance of liver metastasis (Figure 2(f)). Hence, this case was interpreted as a CR. The patient did not experience any adverse events due to S-1 administration and irradiation. No sign of recurrence or metastasis was noted 18 months after CR was confirmed.

## 3. Discussion

S-1 is an oral anticancer agent containing tegafur, a metabolically activated prodrug of 5-FU, and 2 biochemical modulators [1]. S-1 is a key drug in treating gastric cancer. S-1 plus cisplatin is considered a standard first-line treatment for advanced gastric cancer in Japan [2, 3]. The ACTS-GC trial demonstrated that adjuvant S-1 chemotherapy should be the standard treatment for stage II/III gastric cancer following gastrectomy with extended lymph node resection [3, 4]. The SPIRITS trial demonstrated that S-1 plus cisplatin was superior to S-1 alone in terms of progression-free survival (PFS) and overall survival (OS) [2]. However, subgroup analyses of the trial demonstrated that the addition of cisplatin had few benefits for elderly patients [2]. The GC0301/TOP-002 trial did not show significant superiority in the case of S-1 plus irinotecan compared with S-1 alone [5]. The JCOG 9912 trial showed cisplatin plus irinotecan was not superior to S-1 or continuous infusion of 5-FU, and that S-1 was noninferior to 5-FU [6]. Therefore, for convenience, oral administration of S-1 could replace intravenous 5-Fu in the treatment of advanced gastric cancer and could be considered a standard first-line treatment [6].

S-1 is usually administrated for 4 weeks, followed by a 2-week drug-free period. Adverse reactions related to S-1 therapy commonly begin to appear 2-3 weeks after treatment starts [7]. The 2-week regimen of S-1 followed by a 1-week drug-free period might mitigate adverse reactions and prolonged medication period [7]. Two phase II studies of the 2-week regimen of S-1 showed equivalent OS and PFS compared with other conventional chemotherapeutic regimens [8, 9]. Our patient did not experience any adverse events during the 15 cycles of the 2-week regimen of S-1.

The liver is a common site of metastasis of gastric cancer; however, the treatment for liver metastasis of gastric cancer has not been well established. The results of metastectomy for liver metastasis of gastric cancer have been disappointing; thus, metastectomy of the liver should be performed in selected patients as part of multidisciplinary treatments [10, 11]. Local-regional radiation plus systemic chemotherapy administered as postoperative treatment was effective for controlling recurrence of gastric cancer [12]. In our case, hepatic radiation was efficacious against liver metastasis. Nakamura reported on the efficacy of hepatic radiation plus systemic chemotherapy, including S-1, for liver metastasis of gastric cancer [13]. Addition of the radiation as salvage might be useful for the patients with liver metastasis of gastric cancer.

In conclusion, we reported a case of suspected liver metastasis that showed CR to S-1 chemoradiotherapy. Thus, S-1 chemoradiotherapy is likely to be effective in treating patients with liver metastasis of gastric cancer. S-1 has recently been approved by the EMA, product name Teysuno.
